# Mature Erythrocytes of *Iguana iguana* (Squamata, Iguanidae) Possess Functional Mitochondria

**DOI:** 10.1371/journal.pone.0136770

**Published:** 2015-09-14

**Authors:** Giuseppina Di Giacomo, Silvia Campello, Mauro Corrado, Livia Di Giambattista, Claudia Cirotti, Giuseppe Filomeni, Gabriele Gentile

**Affiliations:** 1 Dipartimento di Biologia, Università Tor Vergata, Roma, Italia; 2 IRCCS Fondazione Santa Lucia, Roma, Italia; 3 Istituto Telethon Dulbecco, Istituto Veneto di Medicina Molecolare, Padova, Italia; 4 Cell Stress and Survival Unit, Danish Cancer Society Research Center, Copenhagen, Denmark; University of Alabama at Birmingham, UNITED STATES

## Abstract

Electron microscopy analyses of *Iguana iguana* blood preparations revealed the presence of mitochondria within erythrocytes with well-structured *cristae*. Fluorescence microscopy analyses upon incubation with phalloidin-FITC, Hoechst 33342 and mitochondrial transmembrane potential (Δψ_m_)-sensitive probe MitoTracker Red indicated that mitochondria i) widely occur in erythrocytes, ii) are polarized, and iii) seem to be preferentially confined at a "perinuclear" region, as confirmed by electron microscopy. The analysis of NADH-dependent oxygen consumption showed that red blood cells retain the capability to consume oxygen, thereby providing compelling evidence that mitochondria of *Iguana* erythrocytes are functional and capable to perform oxidative phosphorylation.

## Introduction

In vertebrates, erythrocytes originate from multipotent hematopoietic stem cells that start a process of differentiation leading to first burst-forming units-erythroid (BFUe), then colony-forming units-erythroid (CFUe), normoblasts, erythroblasts, reticulocytes, and ultimately mature erythrocytes [1]. In mammals, during erythroblast maturation, erythroblasts normally lose their nucleus and mitochondria. In fact, in mammals, nuclear retention in erythroblasts is associated with specific conditions or pathologies [2]. In a traditional view, mammals’ anucleate-erythrocyte condition would be considered as an evolutionary response to the need to optimize surface/volume for hemoglobin and the need to cope with a circulatory system that presents end-blood-vessels smaller than in other vertebrates [3]. Flexibility and augmented capability of big nucleated erythrocytes to squeeze through small capillaries would be derived from the extrusion of the nucleus and also other cellular organelles, such as endoplasmic reticulum, not required for erythrocytes’ main function as oxygen carriers. However, such a theory has recently been questioned and evidence has been provided showing that regulation of cytoskeleton for deformation into the biconcave shape may not necessarily require nuclear extrusion. Instead, the extrusion of both mitochondria and nucleus may help mammal erythrocytes against oxidative stress caused by high-sugar and high-heme conditions [4]. Moreover, the extrusion of both mitochondria and nucleus could in general be beneficial for erythrocytes as both source and target of damage derived from the generation of reactive oxygen species (ROS) would be eliminated. As mitochondria consume oxygen during oxidative phosphorylation, their extrusion during erythrocyte maturation should also increase the efficiency of oxygen transport in mature erythrocytes. Thus, in this perspective, loss of mitochondria could benefit erythrocytes of all vertebrates. Such a hypothesis has been recently tackled and contrasted by Stier and collaborators [5] who demonstrated the presence of functional mitochondria in zebra finch erythrocytes and found that levels of oxidative stress in the blood of zebra finches are not higher than those observed in a species of mammal of comparable size.

While it is known that nucleus is present in erythrocytes of all vertebrates, with a few exceptions including mammals, it is less clear to what extent mature nucleated erythrocytes in vertebrates also possess functional mitochondria. Importantly, Stier and collaborators [5] applied a multiple approach to investigate the presence of functional mitochondria in mature nucleated erythrocytes in birds, as prior studies, based on biochemistry or ultra-morphology, had led to incomplete results [6–10]. Direct and indirect evidence has been cumulated for fish, showing that functional mitochondria occur in erythrocytes of several species [11–15]. In amphibians, mitochondria were found close to the nuclear envelope in mature erythrocytes of the urodele *Triturus cristatus* [16] and few anuran (*Rana*) species [7,17]. For the last, indirect evidence of functional mitochondria was also provided [6,18]. For reptiles, Yasuzumi [19] stated that mitochondria were found close to the nuclear envelope in mature erythrocytes of turtles, although no image was provided. Indirect evidence was instead offered to support the existence of functional mitochondria in erythrocytes of a viperid snake [20] and an agamid lizard [21].

Here we used electron and fluorescence microscopy to document the existence of mitochondria in the cytoplasm of mature erythrocytes of *Iguana iguana* (Squamata, Iguanidae). We also used a Clark-type oxygen electrode to demonstrate electron flow to oxygen as a result of oxidative phosphorylation in *Iguana* erythrocytes.

## Materials and Methods

### Blood collection and fractionation

Blood (2 mL) was drawn from the caudal vein of 2 adult green iguana (*Iguana iguana*) males and 2 adult females hosted at the BIOPARCO of Rome (Italy). Iguanas were not related by kinship. Blood samples were obtained using heparinized syringes and stored in ice after collection. Samples were then immediately transported to the laboratories of the Department of Biology (Tor Vergata University) for subsequent analyses. As soon as in the laboratory, whole blood was fractionated by centrifugation at 1500 *g* for 12 min at room temperature with gently acceleration and deceleration to avoid mixing different phases. This methodology allows separate the blood into an upper plasma layer, a lower red blood cell layer, and a thin interface containing the white blood cells (buffy coat). Plasma and, a later stage, also leucocytes were aspirated off from the top of the tube, while erythrocytes were collected by the bottom to avoid any even little contamination. Erythrocytes thus prepared were next used for different analyses.

### Ethic statement

Animal manipulation and blood sampling were performed according to a protocol that minimized animal stress, in accordance with the European Community guidelines and with the approval of relevant National (Ministry of Health) and local (Institutional Animal Care and Use, Tor Vergata University; BIOPARCO Foundation) ethical committees.

### Erythrocyte mitochondrial DNA amplification

In order to demonstrate the presence of mitochondrial DNA in *Iguana* erythrocytes, we extracted DNA from purified erythrocytes of each of the four iguanas using the QIAamp DNA Mini Kit (Qiagen). We PCR amplified and sequenced a mitochondrial DNA target 903-bp region including 709 bases of the 3’ end of the *ND4* subunit of the nicotinamide adenine dinucleotide dehydrogenase gene and the tRNA genes histidine, serine, and leucine (in part). Primers and PCR conditions were as used by Malone and collaborators [22]. Sequences were deposited in Genbank (accessions KT326936, KT326937, KT326938, and KT326939). To safely exclude amplification of nuclear copies of mtDNA (numts) we compared our sequences with the corresponding functional sequence (accession AJ278511) obtained from DNA extracted from heart and liver [23]. Sequences were aligned by using MUSCLE ver. 3.8.31 [24]. We used DNASP [25] to calculate the number of synonymous, non-synonymous, and stop-codon substitutions between sequences.

### Electron microscopy evaluation of erythrocyte ultrastructure

Erythrocyte ultrastructure was obtained by transmission electron microscopy (TEM). Red blood cells were separated and fixed with 2.5% glutaraldehyde with 0.1 M sodium phosphate, pH 7.4 for 1 hour at ice temperature. Sample were post-fixed with osmium tetroxide, stained with uranyl acetate, dehydrated in ethanol and embedded in Epon resin. After sectioning, samples then were collected on uncoated nickel grids and observed.

### Fluorescence microscopy analyses

The mitochondrial transmembrane potential (Δψ_m_) of iguana's erythrocytes was analyzed taking advantage of the Δψ_m_-sensitive probe MitoTracker Red CMXRos by fluorescence microscopy. In detail, *Iguana* blood samples were diluted in PBS (1:5 v/v) and stained for 30 min with 50 nM of MitoTracker Red CMXRos. This is a derivative of X-Rosamine that enters the cells due its lipophilic nature and accumulates into functional mitochondria driven by Δψ_m_. Once in the organelles, the probe is very well retained also upon fixation with paraformaldehyde and, at variance with other probes (e.g., Mito Tracker Red FM, or Green FM), is recommended by the manufacturer for applications in fixed cells, and for multicolor labeling experiments. Indeed, Mito Tracker Red CMXRos red fluorescence is well resolved from the green fluorescence of other probes. Afterwards, blood was smeared on a d-polylisinated slide, fixed with 4% paraformaldehyde for 10 minutes and permeabilized with 0.2% Triton X-100. Each step, blood smear was gently washed three times with cold PBS to prevent any loss of the cells. Next, FITC-conjugated phalloidin (1:1000) was added to the blood smears and incubated at RT for 40 min to visualize actin fibers, that especially in the erythrocytes, are mostly confined underneath the cell membrane. Hoechst 33342, that we used to stain DNA (and therefore identify the cell region occupied by nuclei), enters equally living and permeabilized cells and strongly bounds dsDNA. Therefore, after washing with PBS, blood smears were incubated with 1mL of Hoechst 33342 (1:10000 in PBS) for 30 min, gently washed three times in PBS and visualized by fluorescence microscopy.

### Oxygen consumption measurement

Oxygen consumption in intact erythrocytes was determined at 25°C using a Clark-type oxygen electrode equipped with thermostatic control and magnetic stirring. Purified erythrocytes obtained from 0.25 mL of *Iguana* blood specimens were re-suspended in the same volume of PBS and added to in 1.25 mL of measurement buffer (125 mM KCl, 10 mM Tris/MOPS, pH 7.4, 10 μM Tris/EGTA, pH 7.4, 1 mM K_2_HPO_4_) upon incubation with digitonin. 5 mM glutamate, 2.5 mM malate and 1 mM NADH were also added to the experimental buffer to sustain complex I-dependent oxygen consumption. In particular, NADH provides the reducing equivalents for the electron transport chain, while malate and glutamate fuel the malate-aspartate shuttle, which is required for the virtual intra-matrix uptake of NADH. At the end of each measurement, 5 μM of complex I (NADH dehydrogenase) inhibitor rotenone was always added to verify that oxygen consumption was effectively driven by complex I. Measurements were also repeated in the presence of catalase at the concentration of 0.5 U in order to rule out any side oxygen consumption due to its partial reduction to H_2_O_2_. Indeed, it is well known that H_2_O_2_ production frequently occurs in erythrocytes starting from one-electron reduction of oxygen by heme-contained iron of hemoglobin. Superoxide produced (spontaneously or by superoxide dismutase-mediated catalysis) dismutates in H_2_O_2_. Therefore, the addition of catalase into the oxygraphic chamber is a tool commonly used to understand whether oxygen is fully reduced by electron transport chain to H_2_O, or only partially to H_2_O_2_. In the first case catalase addition is basically ineffective and it does not change oxygen consumption rate. By contrast, when hydrogen peroxide is produced by any source, catalase rapidly dismutates H_2_O_2_ into water and molecular oxygen through the reaction

$$2{\text{H}}_{2}{\text{O}}_{2}\;\to {\text{ H}}_{2}\text{O }+{\text{ O}}_{2}$$

In accordance with this reaction, for every 2 moles of H_2_O_2_, catalase re-generates 1 mole of O_2_, this increasing oxygen concentration inside the oxygraphic chamber and virtually decreasing oxygen consumption up to half, case in which all the available oxygen is reduced to H_2_O_2_ and not to H_2_O by the mitochondrial respiratory chain.

### Statistical analyses

We used the Wilcoxon-Mann-Whitney test of a difference between two groups to test the null hypotheses that oxygen consumption was not different between: i) solutions containing only buffer (no treatment) and solutions containing buffer and erythrocytes (treatment 1); ii) solutions containing only buffer (no treatment) and solutions containing buffer and erythrocytes + catalase (treatment 2). We also used the Wilcoxon signed-rank test for matched pairs to test the null hypothesis that oxygen consumption was not different between treatments 1 and 2. As statistical power is affected by the size of the effect and the size of the sample used to detect it, we performed a post-hoc analysis of achieved power (1-β) for each test, given α = 0.05, N_sample1_ = 4, N_sample2_ = 4, and effect size (*d* and *d*_z_), with correlation (*r*) between treatments 1 and 2 being equal to 0.85. In a conservative approach, we estimated power of tests using the minimum asymptotic relative efficiency method for two-tailed tests, as implemented in G*Power ver.3.1.9.2 [26].

Remaining statistical analysis was performed by using Past ver. 2.16 [27].

## Results and Discussion

The four mitochondrial DNA sequences obtained were between 95% and 96% similar to the sequence used as functional reference. No stop codon substitutions were observed in none of the four coding sequences analyzed, while the maximum number of synonymous and non-synonymous substitutions between sequences was 26 and 4, respectively. The maximum number of synonymous and non-synonymous substitutions between sequences increased to 30 and 5, respectively, when the reference sequence was included in the analysis. This is strongly suggestive that, for all the four iguanas, we amplified a functional copy of the ND4 gene from erythrocyte mtDNA.

**Fig 1:**
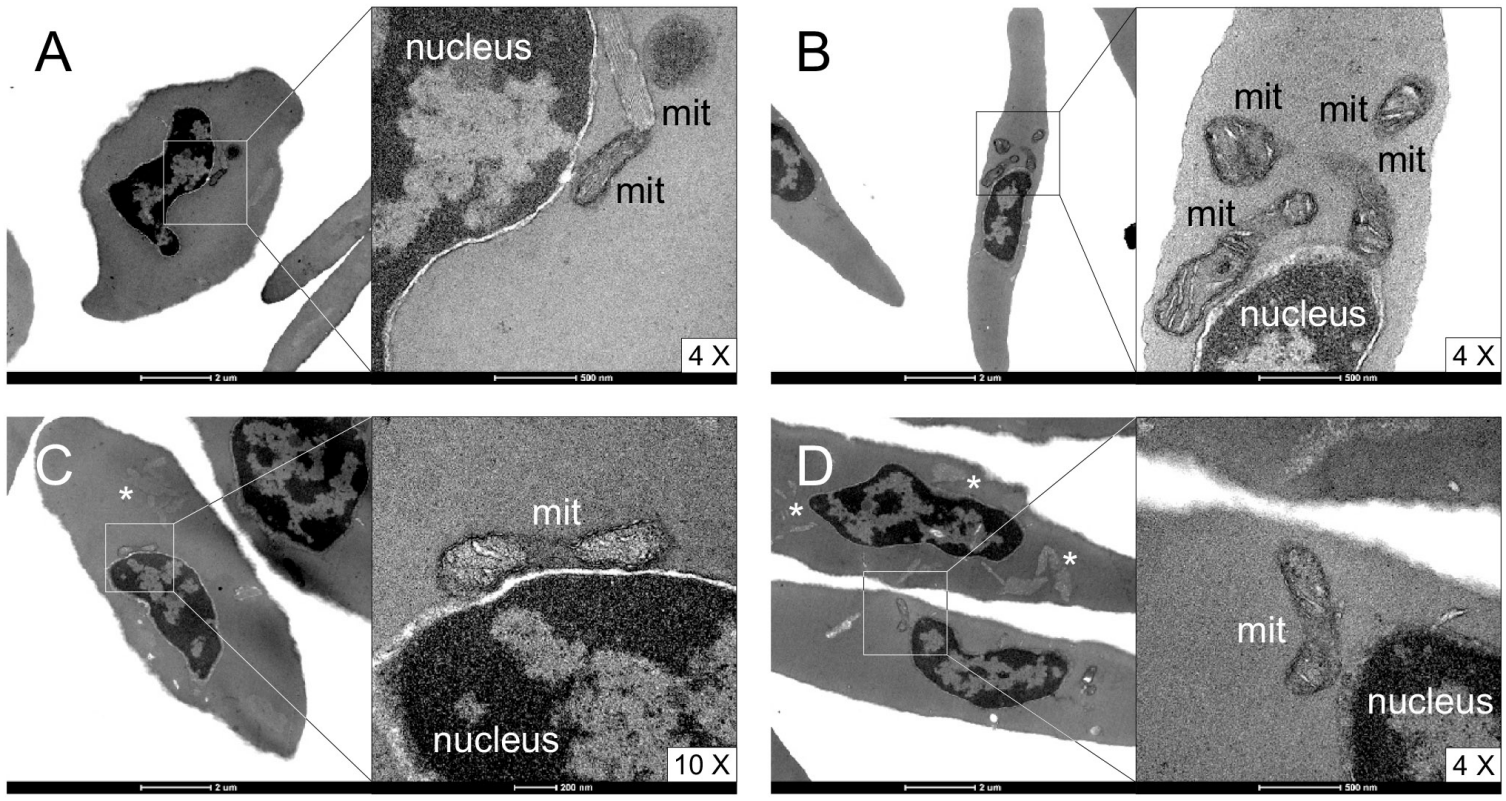
Ultrastructure of *Iguana* erythrocytes. (A-D) Four different fields of blood specimens containing *Iguana* erythrocytes. Nuclei, mitochondria (mit) and uncharacterized internal membrane pools resembling endoplasmic reticulum (*) are indicated. High magnification images (4 and 10X) are shown on the right of each picture.

In order to assess whether erythrocytes from *Iguana iguana* contain mitochondria, we first performed electron microscopy analyses of blood preparations to visualize ultrastructure details of red blood cells. Fig 1 shows representative images of nucleated erythrocytes displaying organelles that did not appear widely distributed in the cytosol, but were present in small numbers, mainly localized at the nuclear border. High magnification images unambiguously showed that those organelles were mitochondria with well-structured *cristae*. However, due to the cut plane, some mitochondria were not included in the section, and only some erythrocytes showed further intra-cytoplasmic structures resembling an undefined pool of internal membranes (e.g., traces of endoplasmic reticulum). Based on these results, which were consistent with prior studies of vertebrate erythrocytes reporting occasional retention of mitochondria and other organelles during the maturation phases [16,28], we could not assess whether mitochondria were regularly or just occasionally present in *Iguana* erythrocytes. In fact, we were still not able to distinguish if they were in a dismantling phase, reasonably during erythrocytes maturation or conversely, whether they were retaining their functionality.

**Fig 2:**
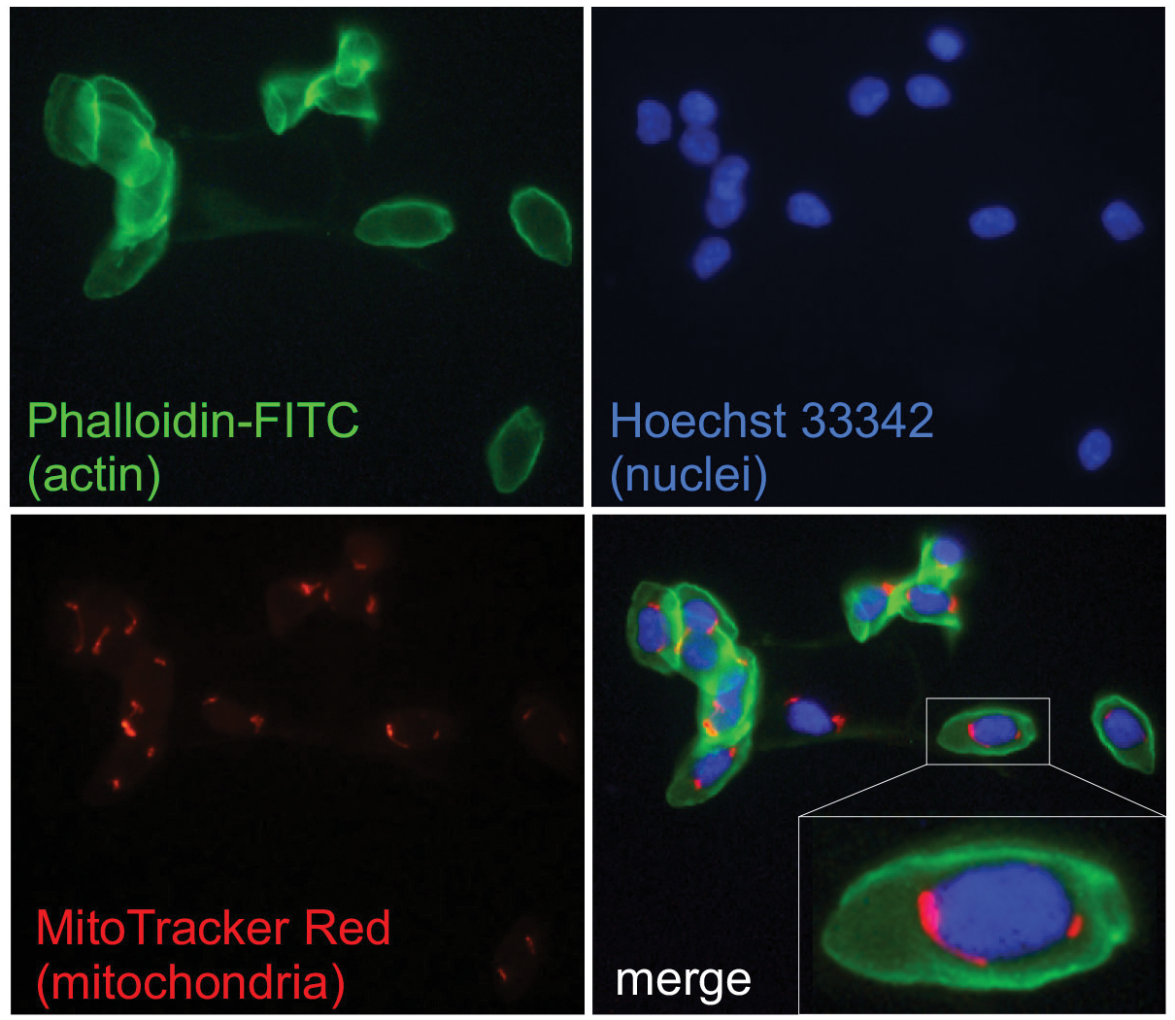
Fluorescence images of *Iguana* erythrocytes. *Iguana* erythrocytes stained with: phalloidin-FITC (green) to visualize actin fibers below the plasma membrane; the DNA-binding dye Hoechst 33342 (blue) to highlight nuclei; the Δψ_m_-sensitive probe MitoTracker Red CMXRos (red) to localize polarized mitochondria. Superimposition (merge) of the three fluorescences is shown at the bottom, along with a 3X magnification detail of one erythrocyte.

To address these issues, we performed fluorescence microscopy analyses upon incubation with phalloidin-FITC, Hoechst 33342 and the mitochondrial transmembrane potential (Δψ_m_)-sensitive probe MitoTracker red CMXRos to highlight cell boundaries, nuclei and mitochondria, respectively. Images shown in Fig 2 unambiguously indicate that mitochondria widely occur in all erythrocytes collected from *Iguana* and they seem to be preferentially confined at a "perinuclear" region, reinforcing the evidence obtained by electron microscopy.

The fluorescence microscopy analyses provided the direct evidence that each iguanas’ erythrocyte has mitochondria, clearly suggesting that, by retaining their Δψ_m_, they are virtually able to generate the proton-motive force required to produce adenosine triphosphate (ATP).

To finally answer this question, we measured NADH-dependent oxygen consumption in iguanas’ erythrocytes by a Clark-type oxygraph. In order to rule out that oxygen consumption was due to any possible side partial reduction to H_2_O_2_ catalyzed by heme iron of hemoglobin (or by mitochondria itself by a futile partial reduction of oxygen), and not driven by mitochondrial electron transport chain, we performed these analyses in the presence or absence of the H_2_O_2_-scavenging enzyme catalase. Indeed, by catalyzing the disproportionation of H_2_O_2_ in O_2_ and H_2_O, catalase regenerates oxygen in the oxygraphic chamber and virtually produces a decrease of the oxygen consumption rate, which will be proportional to the concentration of H_2_O_2_. Raw data are presented in Table 1.

**Table 1:** Oxygen consumption of *Iguana* erythrocytes.

Individual	Buffer	RBC	RBC + rot	RBC + cat
**E1**	0.160	0.575	0.195	0.515
**E2**	0.060	0.545	0.120	0.420
**E3**	0.015	0.770	0.230	0.465
**E4**	0.045	1.005	0.135	0.695

Individual: identification code for each iguana used in this study.

Buffer: measurement buffer without red blood cells.

RBC: red blood cells.

RBC + rot: red blood cells incubated with rotenone.

RBC + cat: red blood cells incubated with catalase.

Data are expressed as nmol of oxygen per minute.

**Fig 3:**
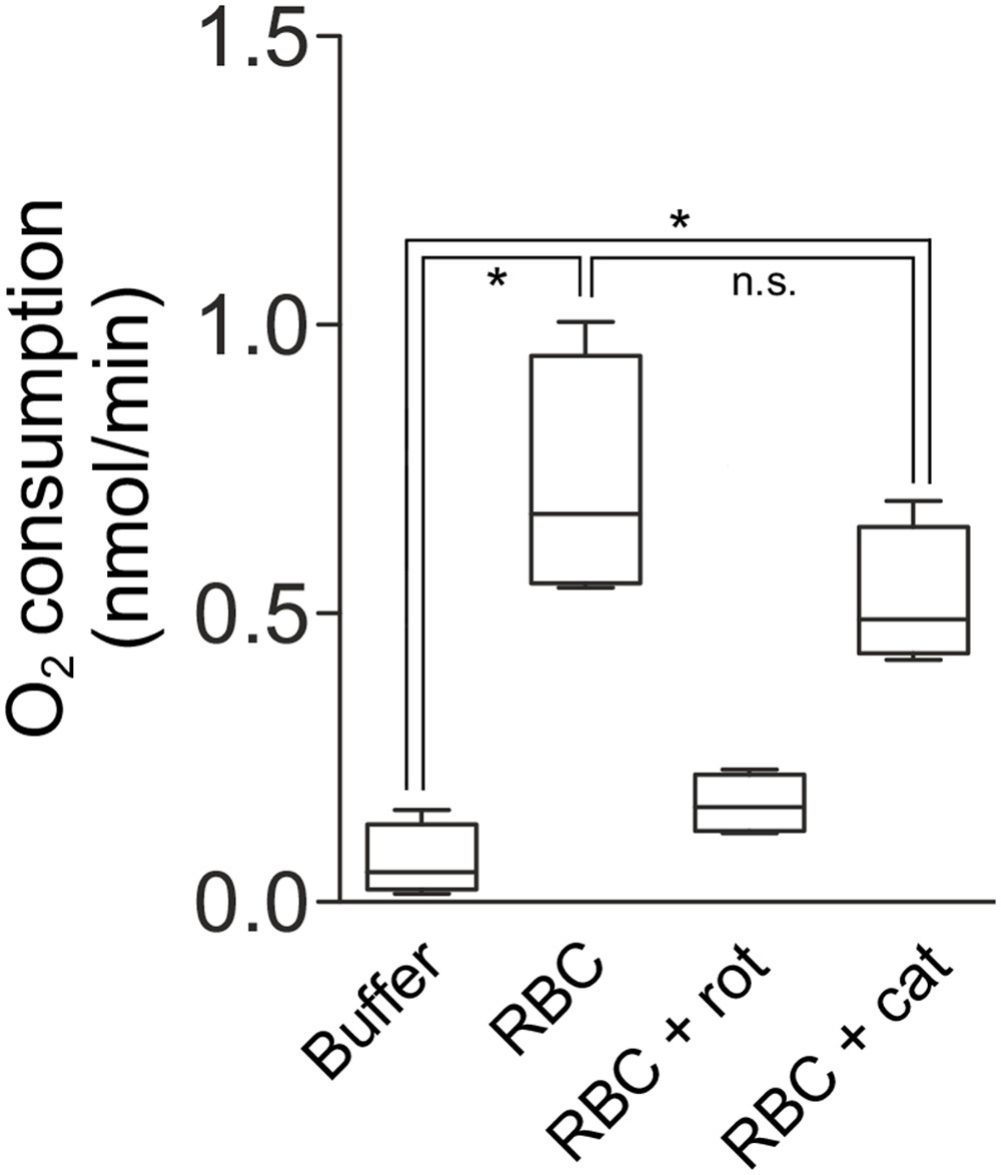
Oxygen consumption of *Iguana* erythrocytes. Purified erythrocytes obtained from 0.25 mL of blood were dissolved in 1.25 mL of measurement buffer containing 2.5 mM malate, 5 mM glutamate and 1 mM NADH to fuel complex I. Upon incubation with digitonin, oxygen consumption was measured for up to 5 minutes in the presence or absence of catalase (0.5 U) and expressed as nmol of oxygen per minute. Five μM rotenone was finally added to the oxygraphic chamber in order to irreversibly inhibit complex I activity and verify that oxygen consumption was dependent on complex I-driven mitochondrial respiration. (Buffer, measurement buffer without red blood cells; RBC, red blood cells; RBC + rot, red blood cells incubated with rotenone; RBC + cat, red blood cells incubated with catalase). *p = 0.030; n.s., not significant (*p* = 0.176).

Values displayed in Fig 3 represent the mean ± SD of single measurements performed on N = 4 individuals. The achieved power was > 0.98 for tests i) and ii), confirming that N_sample1_ = 4 and N_sample2_ = 4 were adequate for our statistical analyses. For the Wilcoxon signed-rank test between treatments 1 and 2, the achieved power was 0.45.

Results show that: i) red blood cells retained the capability to consume oxygen, and ii) catalase did not significantly modulate oxygen concentration, suggesting that this was not (or only moderately, in case we made a Type II error) associated with a side-production of H_2_O_2_, but reasonably due to mitochondrial activity.

Energy is required by erythrocytes to sustain several physiological functions and, with regard to the energetic metabolism—although with differences among taxa—nucleated mature erythrocytes have been reported to be metabolically more active than mammalian anucleated ones [29]. Besides glycolysis, functional oxidative phosphorylation could guarantee further energy to drive several energy-requiring processes and provide metabolites capable to modulate respiratory physiology [9]. In fact, among the potential roles of mitochondria inside nucleated erythrocytes, recently discussed by Stier and collaborators [5], it has been shown that the rate of ATP production by oxidative phosphorylation modulates hemoglobin affinity to oxygen in snake erythrocytes [20]. In particular, it has been reported that, under hypoxic conditions, the concentration of nucleoside triphosphates (primarily ATP) decreases with a trend inversely correlated with oxygen affinity [30]. Bartlett [31] reviewed the occurrence of possible hemoglobin-affinity modifiers − i.e. red-cell organic phosphates (RCOP) − in different vertebrate species including *Iguana iguana*, with emphasis on 2,3-diphosphoglycerate (DPG), inositol pentaphosphate (IP_5_), ATP and guanosine triphosphate (GTP). Interestingly, out of the four compounds analyzed, ATP resulted highly predominant in *Iguana* erythrocytes. Additionally, it has been shown that *Iguana* hemoglobin possesses structural requisites for ATP binding [32]. Admittedly, other factors also contribute to affect oxygen-affinity of hemoglobin in reptiles with adaptation to hypoxia, including temperature, other allosteric interactions with cellular effectors, and gene-based changes in hemoglobin structure and intrinsic oxygen-binding [33–35]. However, the role of oxidative phosphorylation, as long as the cellular mechanisms responsible for decreases in ATP concentration during hypoxia still await an in-depth investigation in reptile nucleated erythrocytes.
